# Implications of causality in artificial intelligence

**DOI:** 10.3389/frai.2024.1439702

**Published:** 2024-08-21

**Authors:** Luís Cavique

**Affiliations:** Universidade Aberta, DCeT and Lasige, FCUL, Lisboa, Portugal

**Keywords:** AI bias, responsible AI, fair AI, explainable AI, causal AI

## Abstract

Over the last decade, investment in artificial intelligence (AI) has grown significantly, driven by technology companies and the demand for PhDs in AI. However, new challenges have emerged, such as the ‘black box’ and bias in AI models. Several approaches have been developed to reduce these problems. Responsible AI focuses on the ethical development of AI systems, considering social impact. Fair AI seeks to identify and correct algorithm biases, promoting equitable decisions. Explainable AI aims to create transparent models that allow users to interpret results. Finally, Causal AI emphasizes identifying cause-and-effect relationships and plays a crucial role in creating more robust and reliable systems, thereby promoting fairness and transparency in AI development. Responsible, Fair, and Explainable AI has several weaknesses. However, Causal AI is the approach with the slightest criticism, offering reassurance about the ethical development of AI.

## 1 Introduction

Technology companies have significantly increased their investment in artificial intelligence (AI) in the last decade. During this period, the demand for AI Ph.D.’s increased substantially, along with the massive acquisition of high-performance computers (McKinsey, 2022). These efforts, backed by billion-dollar investments, have brought AI into the spotlight.

Media exposure has also brought significant challenges to AI, including its ‘black box’ nature and bias. The ‘black box’ refers to the difficulty in understanding the decisions made by complex AI models, making them opaque and difficult to interpret. Furthermore, bias in AI occurs when models reproduce or amplify existing biases in training data, leading to unfair decisions that can negatively affect individuals and groups. Below, we present three confirmed cases of bias in AI:

- In 2015, Google’s image recognition algorithm wrongly labeled African American people as ‘gorillas’. After discovering the error, Google publicly apologized and worked to fix the problem.
- In 2018, Amazon faced an issue with its recruiting AI system, demonstrating a bias against women. The cause of the bias was in the training data used, which consisted predominantly of male candidate resumes submitted to Amazon over 10 years. The system was eventually discontinued.
- Angwin et al. (2016) criticize the COMPAS software, used in the United States to assess the risk of recidivism of prisoners, for bias against African Americans. Given the confusion matrix (relapsed, did not re-offend) versus (low risk, high risk), the False Positive corresponds to (did not re-offend, classified as high risk) and the False Negative to (recurred, classified as low risk). False Negative values are lower among white people than among African Americans. On the other hand, False Positives are higher in white people than in African Americans. The investigation concluded that with comparable criminal histories, the COMPAS system indicates that African Americans are more dangerous.

Several approaches have been developed to overcome the bias problem. Responsible AI seeks to ensure that AI systems are designed and used ethically, considering social impact and principles of justice. Fair AI aims to identify and correct algorithms’ biases to ensure equitable decisions. Explainable AI focuses on creating models that allow users to understand and interpret system decisions, increasing transparency.

Finally, Judea Pearl’s causal revolution (Pearl and Mackenzie, 2018) gave AI a new lease of life. Causal AI uses methods to identify cause-and-effect relationships, providing a deeper understanding of the decisions made and allowing the creation of more robust and reliable systems. Together, these solutions help build fairer and more transparent AI.

## 2 AI approaches against bias

**Figure 1:**
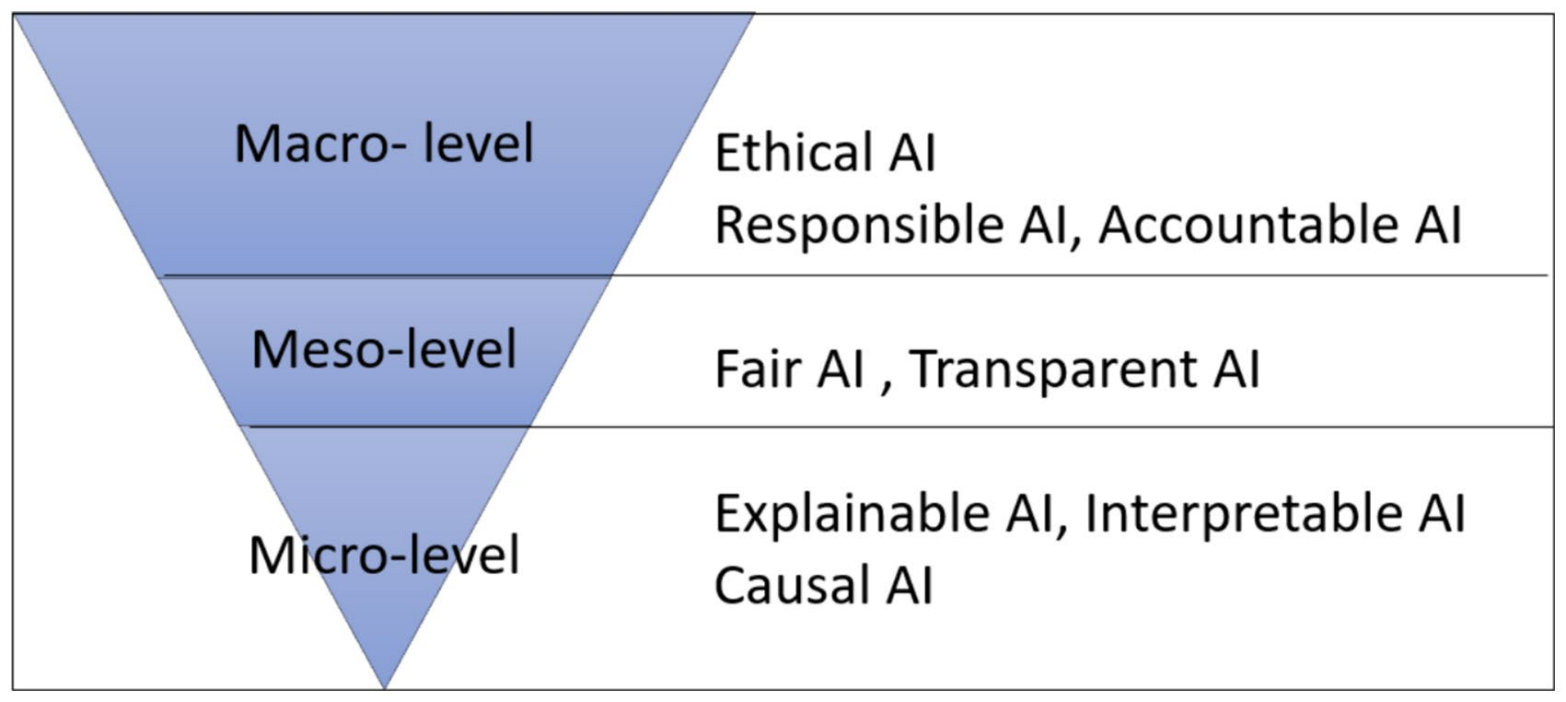
Levels of abstraction to address bias in AI.

Figure 1 presents a holistic view with four approaches in different levels of abstraction (macro, meso, and micro) to combat bias in AI: Responsible AI, Fair AI, Explainable AI, and Causal AI. This work extends the concepts discussed in Cavique (2023).

The macro level of abstraction focuses on legislation and regulatory efforts that support Responsible AI. On the other hand, the micro level of abstraction involves the techniques, tools, and human-computer interaction that support Explainable AI and Causal AI. The meso level combines fundamental regulations and basic techniques, providing a foundation for Fair AI, where the sociological concept of equity is addressed.

Combating bias in AI extends beyond the algorithms. It involves initiatives in legislation, sociology, and human-computer interaction. This interdisciplinary approach promotes comprehensive, legally sound, socially responsible, and user-friendly solutions.

### 2.1 Responsible AI

During her talk, Dignum (2019) humorously highlighted how AI is utilized differently in various regions, such as the United States, China, and Europe. AI is predominantly employed in the United States for commercial purposes and revenue generation. In China, the government has heavily invested in AI for social control and surveillance. Meanwhile, the European Union (EU) has adopted a more cautious approach, emphasizing regulatory frameworks to address ethical, legal, and privacy concerns.

Responsible AI involves the ethical and accountable development and use of AI technologies. In Europe, the goal is to balance fostering innovation with ensuring transparency in AI. Since 2018, the EU has reinforced this commitment by implementing the ‘right-to-explanation’ in algorithmic decision-making (European Commission, 2020).

The EU’s regulatory efforts have led to its advantage over many other countries in a phenomenon known as the Brussels Effect (Bradford, 2012). This effect occurs when other nations adopt the EU’s regulatory decisions. Notable examples include Facebook’s global application of the General Data Protection Regulation (GDPR) and the influence of the European Union’s Emissions Trading System on aviation services and industries.

Following the GDPR, the AI Act was created. The AI Act is European Union legislation establishing rules for using artificial intelligence and adopting a risk-based approach. The law ensures that AI respects fundamental rights, is safe and transparent, and promotes responsible innovation. Passed in 2024, the AI Act could set a global standard for AI regulation in the EU (European Parliament, 2024).

In the United States, several companies advocate acceptance rather than regulation. Every day, new examples continue to emerge. The Responsible Artificial Intelligence Institute (RAI Institute) offers an independent certification program for responsible AI systems, and the TRUSTe Responsible AI Certification is designed to address growing concerns over AI governance.

Regulation advocates call for government intervention to set clear rules for AI to mitigate risks and ensure ethical standards. Certification proponents prefer a flexible, industry-led approach, fearing regulation might stifle innovation and support voluntary adherence to best practices.

### 2.2 Fair AI

We introduce protected attributes and the dichotomy between equality and equity as a foundation for studying fair AI.

Some countries have laws that protect specific groups of people from discrimination based on certain individual attributes, known as ‘protected attributes’. These attributes include race, religion, gender, marital status, age, and socioeconomic stratum. One of the approaches to fair AI is ‘fairness through unawareness’ (Kusner et al., 2017), which removes any of the aforementioned protected attributes from the model.

Equality is treating everyone equally and providing equal opportunities and resources without distinction. Equity, on the other hand, involves recognizing individual differences and offering resources adjusted to each person’s needs to achieve a result equal to others. While equality focuses on uniformity, equity aims to correct pre-existing inequalities, offering more resources to those most in need to ensure everyone can achieve a similar result.

Equality and equity correspond to two distinct notions of justice. Equality is associated with Individual Justice, while equity is associated with Group Justice. Both approaches promote social justice but act differently and correspond to two different ways of seeing the world (Stoyanovich, 2020).

Demographic parity is a broad concept that encompasses overall fairness and equity for different demographic groups. It measures statistical parity used to evaluate and ensure equity in the context of favorable outcome rates from an AI system (Kusner et al., 2017).

Implementing demographic parity, like quotas for less represented groups, can cause reverse (or positive) discrimination and increase development costs. The problem of justice in the equality versus equity dichotomy goes beyond the quality of the data and the AI itself (Raghavan, 2023).

### 2.3 Explainable AI

The publication of ‘The Book of Why’ by Pearl and Mackenzie (2018) sparked a growing interest in understanding machine learning algorithms. The emphasis on interpretability has shifted toward white-box models, including decision trees, decision rules, and linear regression. Reciprocally, methods for explaining predictions from black-box models, such as neural networks, have gained attention, utilizing tools like SHAP and LIME for individual prediction clarity (Molnar, 2024).

Belle and Papantonis (2021) offer a detailed taxonomy for explainable AI (xAI), categorizing (i) algorithms into transparent and opaque and (ii) models into agnostic and specific. They also outline various techniques, including feature relevance explanations, local explanations, model simplifications, and visual explanations.

Model-agnostic techniques can be applied universally across different machine learning models, providing insights without needing in-depth knowledge of the model’s structure. In contrast, model-specific techniques are tailored to particular model types, leveraging their unique attributes to deliver explanations.

Explaining opaque algorithms involves reducing input data (features or instances) or simplifying the model. Fundamental techniques in this domain include:

- Feature relevance: This method assesses the impact of each input feature on the model’s output, highlighting the most significant features used in predictions. An example is SHAP (SHapley Additive exPlanations).
- Local or counterfactual explanations: This approach, known as sensitivity or what-if analysis, creates alternative input scenarios that yield different outputs. It identifies key factors influencing the model’s decisions and tests its robustness.
- Model simplification involves training a more interpretable, simplified model on a subset of the data to mimic the original model’s behavior in specific areas. LIME (Local Interpretable Model-agnostic Explanations) is an example.

Malizia and Paternò (2023) discuss the challenges of xAI in providing transparent AI decision-making. However, many current xAI methods, such as SHAP saliency maps and LIME, often fail to provide understandable explanations for non-technical users. The authors advocate an interdisciplinary approach, integrating knowledge from ethics, law, sociology, and human-centered design to create understandable AI that serves diverse stakeholders.

### 2.4 Causal AI

Judea Pearl’s criticism of traditional AI focuses on its inability to understand and utilize causal relationships (Pearl and Mackenzie, 2018). Pearl argues that traditional AI, much like conventional machine learning, primarily relies on statistical correlations rather than causal inference, which limits its effectiveness and interpretability.

Let *T* → *Y* represent the relationship between cause (*T*) and effect (*Y*). The covariate *X* = {*a*, *b*, *c*}, and the variables *T* and *Y* are represented in the Direct Acyclic Graph (DAG) in Figure 2.1. The goal is to control *X* and study the effect of *T* on *Y*.

**Figure 2:**
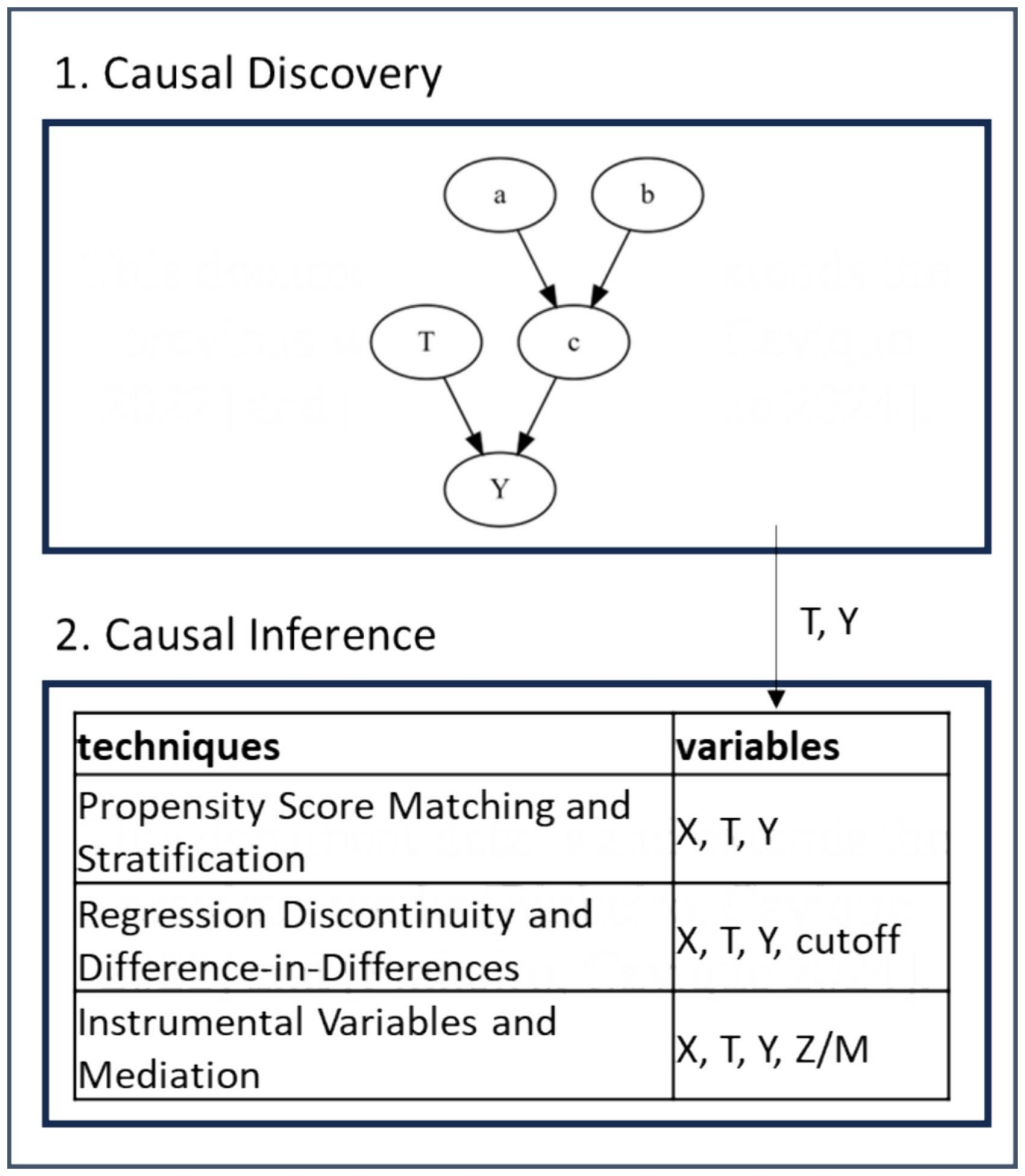
Causal AI framework.

The intervention corresponds to an experimental trial with randomly chosen test and control groups (RCT, ‘random controlled trial’). When RCT is unavailable, the analysts resort to observational data in the company databases. The intervention answers to causal questions, such as:

- ‘what is the effect of *T* = 1 on *Y*?’ (what if *T* = 1?)
- ‘why does *Y* = 1 occur?’ (why does *Y* = 1 occur?)

In causality, we distinguish two fundamental tools, causal discovery and causal inference, shown in the Causal AI framework in Figure 2, where causal discovery and causal inference work sequentially. Causal discovery techniques can help identify possible causal relationships between variables, which can be used as inputs to causal inference models. In causal discovery, the identification of *Y* and *T* is crucial. Causal inference, on the other hand, relies on the DAG and experimental data to estimate causal effects. Causal inference focuses on evaluating the causal impact of the treatment variable *T* on the potential outcome *Y*, considering the causal structure already known in advance. Figure 2.2 lists three types of tools for causal inference.

The effect of treatment (*T*) on outcome (*Y*) can be expressed as the difference between the potential outcomes when the treatment is applied (*Y*^1^) and when it is not applied (*Y*^0^). The difference is the Average Treatment Effect (ATE), generally using the expected value notation: ATE = *E*[*Y*^1^ − *Y*^0^]. The seemingly straightforward expression is more intricate than expected due to the Fundamental Problem of Causal Inference and other issues, like confounders associated with DAGs, paradoxes in interventions within strata, and the counterfactuals of causal inference.

Most of the works in causal discovery are developed by AI practitioners (Pearl and Mackenzie, 2018). Causal inference draws from several scientific areas, including statistics, epidemiology, econometrics, and computer science. To exemplify AI contributions, the work of Athey and Imbens (2016) highlights that Causal Machine Learning can be particularly valuable in identifying treatment effect variations, specifically those associated with observable covariates.

Understanding causal relationships is vital for minimizing bias in AI systems. Recent research by Belle and Papantonis (2021) and Masís (2021) highlights the importance of causality in AI. The goal is to offer clear, transparent, and fair explanations for AI model predictions. Integrating causality into AI can help identify and mitigate biases, leading to more interpretable outcomes.

The relevance of causality extends beyond academia, as many businesses seek actionable insights that are both explainable and free from bias. Notably, Gartner has recognized causal AI as an emerging technology in its 2023 Hype Cycle for New Technologies alongside generative AI.

Causal applications, such as uplift modeling and personalized medicine, use data to make better decisions by understanding cause-and-effect relationships. Uplift modeling estimates the incremental impact of interventions to identify individuals who will respond if, and only if, they are contacted (Pinheiro and Cavique, 2022).

The Causal AI Conference 2024, organized by causaLens, is an event aimed at business and technology professionals interested in applying artificial intelligence in causality analysis. Guests include Turing Award winner Judea Pearl, known for her contributions to Bayesian networks and causal inference, and Guido Imbens, a Nobel Prize-winning economist specializing in econometric methods for causal inference.

### 2.5 Causal AI: challenges and opportunities

Pearl and Mackenzie (2018) mention that in the 1980s, the AI field was divided between two groups: the ‘neats’, who wanted transparent systems with stable behavior, and the ‘scruffies’, who just wanted something that worked. As expected, Pearl considers himself ‘neat’. This narrative introduces the challenges in Causal AI. The first challenge in Causal AI is the ‘scruffies’, who are fascinated by their predictions’ performance and neglect the data’s meaning.

Despite the challenges, Causal AI offers valuable opportunities. Causal AI involves a shift in perspective by creating new questions (using what-if and why) and finding answers that measure the effect of treatment variables, going beyond the classic machine learning prediction.

## 3 Discussion

Artificial Intelligence (AI) bias results in unfair and discriminatory decisions, perpetuating bias toward underrepresented groups. To combat bias in AI, we presented four approaches at different levels of abstraction: Responsible AI, Fair AI, Explainable AI, and Causal AI. By integrating legal frameworks, sociological insights, and user-centered design principles, we can better address the challenges in AI.

Responsible AI is about ethical and transparent technology governance, remaining at an impasse over the best path forward: regulation or certification. Fair AI grapples with issues of justice, such as the dichotomy of equality versus fairness, which go far beyond AI systems (Raghavan, 2023). Explainable AI (xAI) struggles to provide transparency and interpretability, necessitating interdisciplinary approaches. However, many current xAI methods, such as SHAP and LIME, often fail to provide understandable explanations for non-technical users (Malizia and Paternò, 2023). So far, Causal AI is the least criticized approach. Moreover, Causal AI is supported by solid and interdisciplinary scientific foundations (Pearl and Mackenzie, 2018), contrasting with xAI.

With Causal AI, we can identify control variables, use causal models, distinguish between correlation and causation, and reduce bias. Causality also allows for dealing with counterfactuals, helping to understand the impact of specific variables and promoting and enabling counterfactual analyses.

## Data Availability

The original contributions presented in the study are included in the article/supplementary material, further inquiries can be directed to the corresponding author.
